# Gastric Adenocarcinoma: Is Computed Tomography (CT) Useful in Preoperative Staging?

**DOI:** 10.4137/cmo.s2641

**Published:** 2009-08-12

**Authors:** Esther Uña Cidón, Isabel Jiménez Cuenca

**Affiliations:** 1 Department of Medical Oncology, Clinical University Hospital, s/n C/Ramón y Cajal, 47005 Valladolid, Spain.; 2 Department of Radiology, Clinical University Hospital, s/n C/Ramón y Cajal, 47005 Valladolid, Spain.

**Keywords:** gastric adenocarcinoma, preoperative staging, computed tomography, (CT)

## Abstract

**Background and Purpose:**

Although multiple studies testing the accuracy of CT in the preoperative staging of gastric adenocarcinoma have been carried out, their results are controversial. Whilst some authors claim that CT is an accurate method for preoperatively staging gastric cancer, others have advocated the contrary. Because of this discrepancy we have retrospectively reviewed preoperative CT findings compared with histopathological results in patients with gastric adenocarcinoma.

**Patients and Methods:**

Seventy-two patients diagnosed with gastric cancer who underwent potentially curative surgery and preoperative staging CT of quality were included in the study. The size, gastric wall thickening, presence of lymphadenopathy, adjacent organ invasion and location of the gastric mass was recorded. Early tumors (T1 and T2) and more advanced tumors (T3 and T4) were grouped together. CT staging was correlated with the final histopathological stage (TNM). The global results were expressed as sensitivity, specificity, positive predictive value (PPV) and negative predictive value (NPV).

**Results:**

Seventy-two cases were included with fifty-five being male and a median age of 67 years (range 33–91). CT correctly identified the location of the tumor in 56 (53% antropyloric, 18% subcardial). Median time from CT scan to surgery was fourteen days (range 2–49). In T detection: T1/T2 and T3/T4 with sensitivity of 70% and 61%. Lymph node involvement: Sensitivity 49%. Overstaged in 47% Understaged in 75%. Specificity of 53%. Nine patients with colon-mesocolon (5 patients) and pancreas (4 patients) invasion. Sensitivity 44% and specificity 96%.

**Conclusion:**

Spiral CT is not an accurate method in predicting preoperative stages in gastric cancer.

## Introduction

Although the incidence of gastric cancer is decreasing worldwide,^1^ it is still the second leading cause of cancer deaths in the world.^2^ An accurate evaluation of the local and distant extent of the disease is essential to select an optimal therapeutic approach. The depth of intramural tumor invasion and spreading beyond the gastric wall, the involvement of lymph nodes and distant metastases are the most important prognostic factors in gastric cancer.^3^ Although the local surgical treatment is the only therapeutic option with a chance of cure, most patients present advanced disease at diagnosis, so they could not be considered suitable for resection. In these patients it could be relevant to have a sensitive imaging tool for detection and thus avoid the morbidity of an unnecessary laparotomy.^4^–^5^

Because the aim of gastric cancer surgery is to excise the primary lesion adequately, it is very important to know the location of this lesion and the tumor margin before any therapeutic decision can be taken. So in this context, the role of computed tomography (CT) in the preoperative staging of gastric cancer has been suggested as an accurate imaging modality for evaluating the extent of primary gastric cancer and nodal involvement of the disease.^6^–^8^ However, reported results comparing preoperative CT with histopathological findings are variable.^9^–^10^

Because of these contradictory results, we retrospectively reviewed the results of preoperative CT scans compared with subsequent histopathological findings in patients with gastric adenocarcinoma. The aim of this study was to assess the accuracy of spiral CT scanning in this context.

## Patients and Methods

### Patients

Between January 2004 and March 2008, one hundred and thirty cases of gastric cancer were reviewed from the tumor registries from our Hospital. Seventy-two patients underwent potentially curative surgery with at least D1 lymphadenectomy and preoperative staging CT were included in this review. To confirm the diagnosis of gastric cancer in all patients, an endoscopic biopsy was performed prior to examination with CT and all tumors were diagnosed as adenocarcinomas of the gastric mucosa. The institutional research committee was consulted before the study began but its approval was not required.

### Computed tomography

In this study, all CT examinations were performed using CT multicuts of 64 detectors LightSpeed VCT^®^ from General Electric according to the protocol for patients with gastric malignancies as provided by the Spanish Society of Radiology. Informed consent had been obtained from every patient prior to the CT examination.

All patients underwent contrast enhanced CT after a five hours fast and all were scanned in the supine position. Sixty minutes before scanning they were asked to drink 150 ml of oral contrast BarioCT^®^ to opacify the stomach and small bowel. Intravenous contrast Omnipaque^®^ 300 mg Iodo/ml was delivered at a rate of 3 ml per second, through a cannula in the antecubital fossa.

Two series of images were carried out. The first one included thorax and liver 30 seconds after the contrast injection and the second one was taken from the diaphragm to the symphysis pubis 65 seconds after the onset of the contrast injection.

At 15 mm intervals, 5 mm slice thickness were taken and then sequential reconstruction of 1,25 mm. All the images were studied with multiplanar reconstructions (sagittal, coronal and oblique) and with MIP technique (Maximum Intensity Projection) to evaluate the tumor’s vascularization and to identify small lungs nodules.

**Figure 1.**
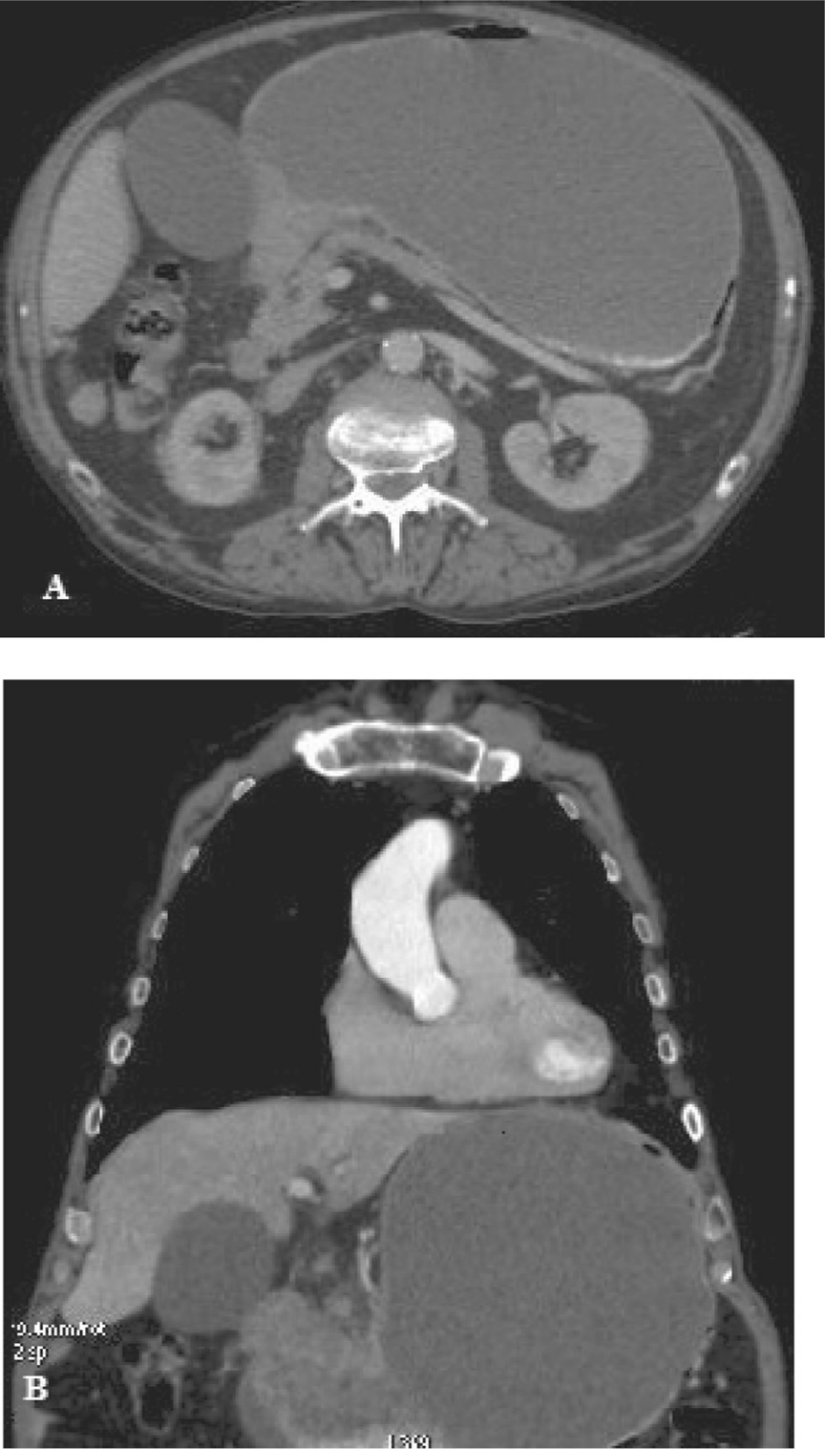
A 71-year-old man with antrum gastric cancer. **A**) Transverse axial contrast-enhanced CT image shows a wall thickening of the gastric antrum. **B**) Coronal reconstruction CT image shows irregularly wall thickening of the gastric antrum.

**Figure 2.**
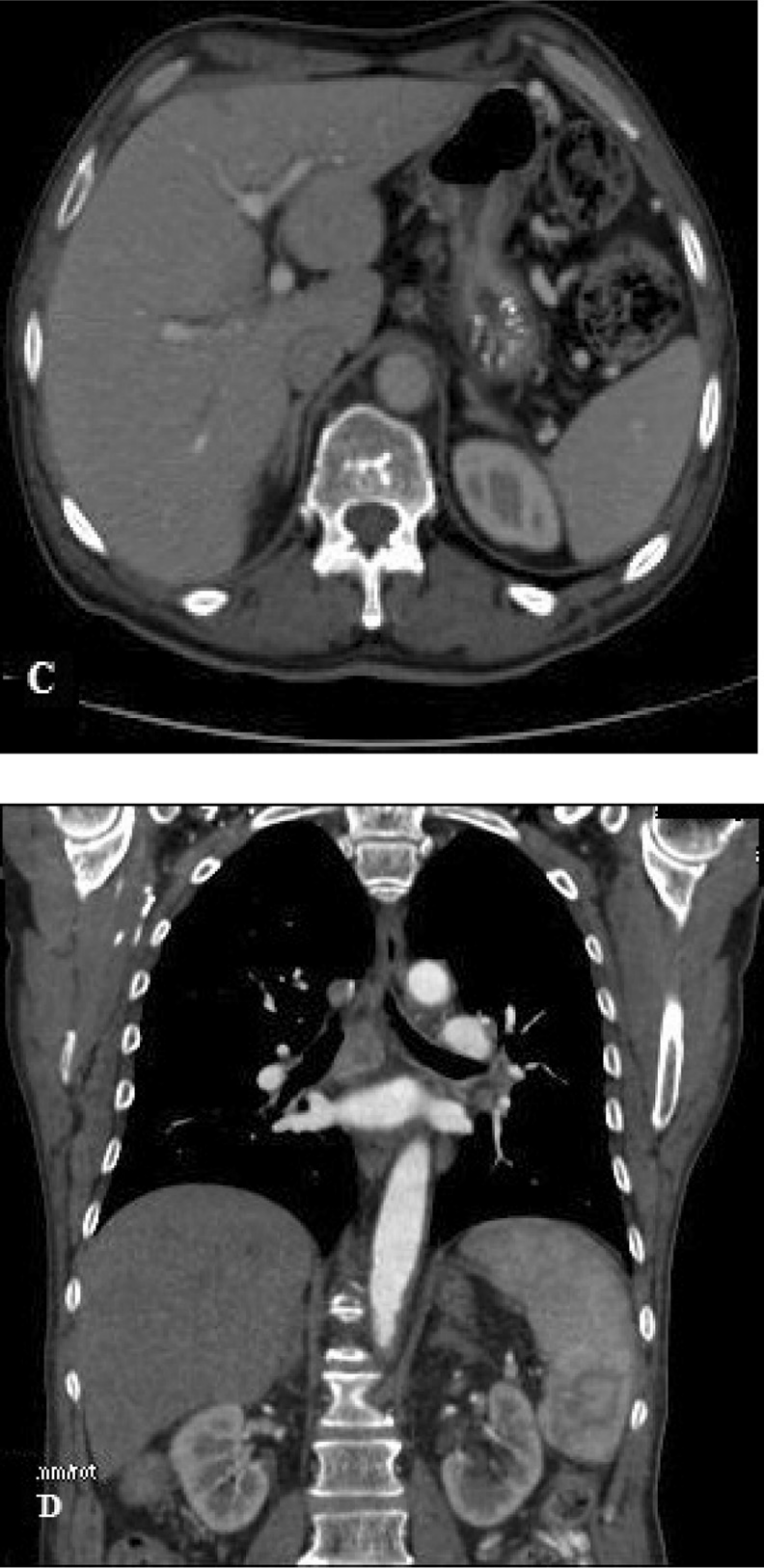
A 57-year-old man with advanced gastric cancer. **C**) A transverse axial contrast-enhanced CT image shows several enlarged lymph nodes along the gastrohepatic ligament. This image shows gastrohepatic ligament lymphadenopathies. Their clinical significance was uncertain. These lymph nodes were pathologically proved to be metastatic lymph nodes. **D**) This coronal reconstruction shows several enlarged lymphadenopathies suggesting metastatic disease which was confirmed by pathological study.

Lymph nodes of a greater size than 10 mm on CT scan were considered malignant (Figure 2).^11^ The CT scans for all patients were considered to be of quality. The size, gastric wall thickening and location of the gastric mass was recorded. On CT scan any lesion considered as malignant showed focal thickening of 6 mm or greater.^3^ Due to the small number of cases involved, early tumors (T1 and T2) were grouped together as were the more advanced tumors (T3 and T4) as the prognosis between these two groups is significantly different. The classification of tumor invasion was as follows; based on previous studies: T3–T4 were classified as such because of the irregular outer border of the thickened gastric wall or perigastric fat infiltration or direct invasion of tumor into a contiguous organs or structures (Figure 1).^3^ The rest of the cases were classified as T1–T2.

The criterion used for concluding direct invasion on CT scans was lack of a fat plane between the gastric mass and an adjacent organ. Invasion of the oesophagus or small bowel was predicted if the gastric mass or wall thickening produced wall thickening of these organs.

In our review we did not include cases where lesions identified on CT scan suggested metastases to the liver and others organs or distant structures such as lymph nodes located in retropancreatic area, paraaortic and mesenteric nodes.

CT staging was correlated with final histopathological stage (TNM).^12^ The gastrectomy tissue and lymph nodes were transferred to the pathology department. Lymph nodes were dissected individually from the surrounding fatty tissue and each node was evaluated independently. The depth of invasion was carefully defined.

Two abdominal radiologists with more than 6 years of experience in abdominal CT imaging performed the image analysis. They were blinded to endoscopic results and macroscopic features and they evaluated CT images at a workstation and gave the diagnosis by consensus. If they did not agree they consulted a third radiologist who gave the definitive diagnosis.

### Statistical analysis

The global results were expressed as sensitivity, specificity, positive predictive value (PPV) and negative predictive value (NPV). These results were defined as follow:^1^

Sensitivity: Number of true positive cases divided by the sum of the true positive cases plus the false negative cases.
Specificity: Number of true negative cases divided by the sum of the true negative cases plus the false positive cases.
PPV: Number of true positive cases divided by the sum of the true positive cases plus the false positive cases.
NPV: Number of true negative cases divided by the sum of the true negative cases plus the false negative cases.

Differences in accuracy for T and N staging were assessed by using the McNemar test. Statistical significance was inferred at a confidence level of 5%. The strength of the agreement between the CT stage and the histopathological stage was determined by the weighted kappa statistic (KS).

## Results

### 

A cohort of seventy-two cases of non-metastatic gastric adenocarcinoma were included in this retrospective study. Fifty-five were males and the median age was 67 years (range 33–91). Gastric primary tumors were detected in all patients on CT scan. In fifteen cases the masses were circumferential. Median time from CT scan to surgery was fourteen days (range 2–49). We detected a high concordance between radiologists in N evaluation (94%), however in 4 patients the collaboration of a third radiologist was necessary. In T evaluation the concordance was 92%.

#### Tumor location

Spiral CT correctly identified the location of the primary tumor in 56 (77%) cases. Spiral CT ascribed an incorrect position in 16 patients. None of these patients had a tumor which could not be visualised on CT. In 53% of cases the tumor was located in antropyloric area (Fig. 1) and 18% were subcardial tumors.

#### T staging

CT scan correctly ascribed as T1/T2 tumors in 7 out of 10 patients with a sensitivity of 70% and identified T3/T4 accurately in 38 out of 62 (sensitivity of 61%) (see Table 1). CT scan findings overstaged the depth of tumor in 30% of patients with histopathological confirmed T1/T2 and understaged 38%. The differences between CT and histopathological findings in T staging were statistically significant (p 0.0001).

#### Lymphadenopathy

Spiral CT correctly identified involvement of lymph nodes in 36 patients giving a sensitivity of 49%. CT findings overstaged the number of lymph nodes involved in 47% and understaged in 75% of cases. However, CT correctly identified 9/17 patients who did not have nodal invasion with a specificity of 53% (Table 1). We have not reported the location of involved lymph nodes.

The differences between CT and pathological findings in N staging were statistically significant (p 0.02).

#### Invasion of adjacent organs

Nine patients had direct invasion of either the colon-mesocolon (5 patients) or the pancreas (4 patients) at histological assessment of the resected specimen. Spiral CT correctly detected such invasion in four patients (S of 44%), but spiral CT provided two false positives with a specificity of 96%.

CT scan identified direct invasion of the pancreas and colon-mesocolonic invasion with a sensitivity of 50% and 67% respectively (Table 1).

## Discusion

### 

This is one of the larger series of patients with gastric adenocarcinoma in whom preoperative CT findings are compared with pathologic findings. There are other studies which have previously evaluated the role of preoperative CT in such patients, but the reported results were variable. Although some investigators claimed that CT could give accurate preoperative staging information,^6^,^7^ others have been less optimistic about the usefulness and accuracy of CT scanning in this context.

In this study, CT was a poor method of identifying lymph node metastases from gastric adenocarcinoma, with a sensitivity of only 49% and poor specifics in the detection of uninvolved nodes with specificity of 53%, both of the results are in agreement with previous authors who have reported sensitivities ranging from 48 to 82% and specificities lower than 62%.^13^–^16^

Although some smaller studies have shown better results, with reported sensitivities approaching 97%,^7^ a prospective study carried out by Cook et al reported a sensitivity for detecting lymphadenopathy of 43% in keeping with our results.^10^ This study detected 14 false negative on spiral CT, five of them were confluent with the primary tumor which was difficult to detect. The inability in detecting lymph nodes adjacent to the primary tumor is a known limitation of CT scan.

The low sensitivity and specificity in detecting metastatic nodes in our study was caused by the presence of metastases in normal-sized or lower than 10 mm nodes and the presence of benign changes as reactive hyperplasia in nodes bigger than 10 mm considered metastatic on imaging criterion. We have obtained a high rate of false negatives (39%), which it could be explained in part by the extended time between CT scan and surgery. True positive results were detected at all lymph node sites, most of them in nodes distant from primary tumor (celiac, retrocrural, or paraaortic lymph nodes were easier to identify). All these findings are important because the presence of disease in these nodal groups identifies advanced disease and would change the therapeutic procedure. In eight patients, enlarged nodes were detected on CT, but they were found to be unaffected in histopathological study (false positive 11%).

The average size of these did not differ from that of the false-positive lymph nodes. Our experience, therefore, suggests that CT is not a good method to differentiate normal lymph nodes from those containing metastases.

In contrast with our results Lee et al^17^ undertook a retrospective study of 67 patients, all of whom underwent radical surgery for gastric cancer before they entered in this study but only 55 patients were eligible to the histopathological T and N analyses. The authors reported a high accuracy in predicting T stage (85,4%) and N stage (81,8%) in the remnant stomach. Although these results are interesting we need to be cautious when interpreting them because the small number of cases they reported and because of the lack of statistical analyses. On the other hand our results are in agreement with Davies et al.^1^ They detected that the ability of spiral CT to identify involvement of N2 lymph nodes considered as nodes far from the primary tumor, was better than for N1 nodes (perigastric nodes), with a sensitivity similar to our results.

Fukuya et al^18^ reported in their study that spiral CT found that the incidence of positive lymph nodes varied with size, with 5% for nodes smaller than 5 mm, 23% for nodes between 10 to 14 mm and more than 80% for nodes greater than 14 mm.

Komaki^19^ compared CT scans with surgical pathologic findings in patients with gastric carcinoma and concluded that lymphadenopathy would be characterized as massive or solitary because massive adenopathies represented metastatic disease in 96% of cases, whereas solitary adenopathy represented metastatic disease in 48% of cases. In our series 75% of the true positive CT examinations showed multiple adenopathy, whereas 25% showed solitary lymphadenopathy. The majority of the false positive examinations (62%) demonstrated only solitary adenopathy.

Dong Ho Lee et al^20^ obtained results similar to ours after their study evaluating spiral CT in staging of gastric cancer. They studied 180 patients diagnosed with gastric cancer and they concluded that CT scan is not accurate to determine the preoperative staging in this type of tumor.

#### Organ

Preoperative knowledge of direct organ invasion is very important in planning the surgical procedure to decide whether surgery is likely to be potentially curative or palliative or whether it may be better to begin with neoadjuvant therapy upfront.

Although in our study the number of patients with adjacent organ invasion was small, we observed that CT failed to detect this in five out of nine patients with histopathological organ invasion.

The sensitivity of CT to detect organ invasion in our series (44%) was very poor although specificity was high (96%), which meant that a positive spiral CT result could be relied upon.

In the report from Davies^1^ et al spiral CT detected 13 of the 17 cases with invasion of colon-mesocolon with a sensitivity of 76%, also demonstrated that spiral CT remains poor (sensitivity 50%) at identifying invasion of the pancreas as previous reports had described (sensitivities varying from 27 to 95%).^13^,^15^ Our study detected better sensitivity in demonstrating colon/mesocolon invasion than pancreatic invasion (67% and 50% respectively) although the number of patients with organ invasion was very small. The reason for this is that the pancreas is an organ that is known to be notoriously difficult to evaluate on preoperative imaging, because of a fat plane between the tumor and the pancreas, which is not a good indicator of invasion. The absence of a fat plane does not necessarily imply invasion. Patients with gastric carcinoma are often malnourished producing a false positive CT finding of pancreatic invasion. Another cause of obliteration of the peripancreatic fat plane is inflammation such as that encountered with pancreatitis.

Differentiation between inflammatory adhesion and true invasion on spiral CT can be very difficult. In this study, we have found one false positive which represented inflammatory changes on histopathological study. Cook et al reported a sensitivity for detection of pancreatic invasion by CT of 60%.^10^

Dehn et al also described four cases in which both CT and surgical findings suggested direct pancreatic invasion but pathologic examination showed only an inflammatory response.^7^

The large number of false positive examinations, combined with only a small number of true positive studies, led to a positive predictive value of only 38% for the CT finding of pancreatic invasion.

In our study all patients were scanned in a supine position, but it has been suggested that prone scanning improves the ability of CT to identify pancreatic invasion.^21^,^22^

Spiral CT has the potential to demonstrate peritoneal and liver metastases because it can be used to examine the entire abdominal cavity but we have not evaluated this in our study.

It has been reported previously that CT is limited in the demonstration of peritoneal metastases, being able to identify peritoneal disease only in the presence of ascites.^23^ CT can identify gross peritoneal disease, with large omental cake-like deposits, but it is vey poor in the detection of isolated nodules due to its limits of resolution.

After all these results it is not surprising that CT scan is considered poor in the prediction of histopathological stage, underestimating it in 55% of patients and overstaging in 13% of patients. This data is in keeping with those of Cook et al^10^ (understaging rate of 51% and overstaging rate of 18%). These results differ markedly from those of Moss et al^6^ who advocated that CT was an accurate method of preoperative staging of gastric carcinoma.

Of the 40 patients overstaged with CT imaging, the mistake was predominantly due to overdiagnosing invasion of lymphadenopathies followed by overdiagnosing of depth of tumor. The causes were the same in the 10 cases of understaging. It is mandatory to be careful in the process of making the decision about the adequacy of surgical assessment because we could overestimate the false positive rate if the operative evaluation was not sufficient for diagnosis of the extent of the disease.

In conclusion, we found that the entire stomach can be visualized by contrast enhanced spiral CT and its findings lead us to consider the process as a good method predicting the location of gastric cancer. However, differentiation between involved and uninvolved lymph nodes was difficult on CT with a relevant rate of false negative and understaging. T staging considering serosal invasion as a border between T1/T2 and T3/T4 by helical CT was markedly better than diagnosis of involvement of lymph nodes. However, taking into account the global results, CT scan was not an accurate tool of preoperative staging when its results have been compared with histopathological findings.

## Figures and Tables

**Table 1.** Spiral CT status of T, lymphadenopathy and organ invasion compared with final histology.

	**Depth of tumor**		**Lymphadenopathy**	**Adjacent organ invasion**
	**T1 and T2**	**T3 and T4**		
Sensitivity	7/10 (70)	38/62 (61)	27/55 (49)	4/9 (44)
Specificity	38/62 (61)	7/10 (70)	9/17 (53)	61/63 (96)
PPV	7/31 (22)	38/41 (92)	27/35 (77)	4/6 (66)
NPV	38/41 (92)	7/31 (22)	28/37 (75)	61/66 (92)
Accuracy	45/72 (62)		58/72 (80)	36/72 (50)

**Depth of tumor (T)**	**Stimated value**	**Confidence interval 95%**	
Sensitivity	0.7	0.42	0.98
Specificity	0.61	0.49	0.73
PPV	0.22	0.08	0.37
NPV	0.92	0.85	1.01
**Lymphadenopathy (N)**			
Sensitivity	0.49	0.36	0.62
Specificity	0.53	0.29	0.77
PPV	0.77	0.63	0.91
NPV	0.29	0.10	0.38

Results are expressed as number follow by (%).

KS for T/N staging with CT in gastric cancer was 0,166; 0,170.
